# *T*_m_-Shift Detection of Dog-Derived* Ancylostoma ceylanicum* and* A. caninum*

**DOI:** 10.1155/2018/7617094

**Published:** 2018-05-13

**Authors:** Yeqi Fu, Mingwei Wang, Xinxin Yan, Auwalu Yusuf Abdullahi, Jianxiong Hang, Pan Zhang, Yue Huang, Yunqiu Liu, Yongxiang Sun, Rongkun Ran, Guoqing Li

**Affiliations:** ^1^Guangdong Provincial Zoonosis Prevention and Control Key Laboratory, College of Veterinary Medicine, South China Agricultural University, Guangzhou 510542, China; ^2^Animal Science Department, Kano University of Science and Technology, Wudil, PMB 3244, Kano 20027, Nigeria

## Abstract

To develop a *T*_m_-shift method for detection of dog-derived* Ancylostoma ceylanicum* and* A. caninum,* three sets of primers were designed based on three SNPs (ITS71, ITS197, and ITS296) of their internal transcribed spacer 1 (ITS1) sequences. The detection effect of the *T*_m_-shift was assessed through the stability, sensitivity, accuracy test, and clinical detection. The results showed that these three sets of primers could distinguish accurately between* A. ceylanicum* and* A. caninum*. The coefficient of variation in their *T*_m_ values on the three SNPs was 0.09% and 0.15% (ITS71), 0.18% and 0.14% (ITS197), and 0.13% and 0.07% (ITS296), respectively. The lowest detectable concentration of standard plasmids for* A. ceylanicum* and* A. caninum* was 5.33 × 10^−6^ ng/*μ*L and 5.03 × 10^−6^ ng/*μ*L. The *T*_m_-shift results of ten DNA samples from the dog-derived hookworms were consistent with their known species. In the clinical detection of 50 fecal samples from stray dogs, the positive rate of hookworm detected by *T*_m_-shift (42%) was significantly higher than that by microscopic examination (34%), and the former can identify the* Ancylostoma* species. It is concluded that the *T*_m_-shift method is rapid, specific, sensitive, and suitable for the clinical detection and zoonotic risk assessment of the dog-derived hookworm.

## 1. Introduction

Hookworms are common intestinal parasites distributed globally, which can cause serious harm to the health of dogs, cats, and human beings. There are four species of hookworms, that is,* Ancylostoma caninum, A. ceylanicum, A. braziliense*, and* Uncinaria stenocephala*, that can infect dogs [1]. According to a recent survey, stray dogs are infected mainly by* A. ceylanicum *and* A. caninum *in China [2]. It was reported that* A. ceylanicum *has become the second most common species of hookworm that can infect humans in Asia [3, 4], especially in Southeast Asian countries such as China [5, 6], Japan [7], Malaysia [8], Laos [9], Thailand [10], and India [11]. Occasionally,* A. caninum *can reach adulthood in people [1]. These two hookworms are zoonotic parasites that can parasitize the host's intestinal tract and cause severe symptoms such as intestinal bleeding, anemia, malnutrition, and dermatitis [12, 13]. Therefore, it is very important to establish a rapid and accurate identification method for the two dog-derived hookworms for the prevention and control of hookworm disease.

Fecal examination is a traditional method for detecting hookworms, but it is easily missed under mild infection. Because of morphological similarities of different hookworm eggs, it is difficult to identify them only by their appearance. Presently, many molecular detection technologies have been applied to the identification of hookworms, such as specific polymerase chain reaction (PCR), multiplex polymerase chain reaction (multi-PCR), restriction fragment length polymorphism (RFLP), and high resolution melting (HRM) [14–16]. However, there are still some shortcomings, such as being unable to detect a large quantity of samples at the same time, complicated operation, and high cost.

Single nucleotide polymorphism (SNP) has become a molecular genetic marker of the third generation after RFLP and microsatellite sequence (MS) [17, 18]. The *T*_m_-shift based on the SNP is a new molecular detection method, where two specific primers and a reverse primer are designed as follows: the 3′-end of specific primer binds to SNP loci, and two GC-rich sequences with unequal length and no pairing with the template are added at the 5′-end, respectively. Then, PCR products of samples are detected according to different peaks of melting curve in different *T*_m_ values [19]. The *T*_m_-shift method can satisfy the high-throughput research and has efficient, accurate, and inexpensive advantages [20]. So far, this method has been applied in many fields including medicine, microbiology, and aquatic biology [21–23]. Recently, we established *T*_m_-shift genotyping method for detection of cat-derived* Giardia lamblia* assemblages A and F [24], which has developed a new genotyping method for veterinary parasitic protozoa.

This study developed a *T*_m_-shift method for detecting* A. ceylanicum* and* A. caninum* from dogs based on three SNP loci of rDNA ITS1 sequences of two hookworms, in order to establish a rapid and accurate method for identifying the species of dog-derived hookworms which could provide a new technical means for clinical detection of hookworms and zoonotic risk assessment.

## 2. Materials and Methods

### 2.1. Source of Samples

Adult* A. ceylanicum* and* A. caninum* samples were isolated and identified by Liu et al. [2, 6], preserved in 75% alcohol, and stored in our laboratory. Fecal samples were collected from stray dogs of pet shelters in Shantou, Foshan, and Shaoguan districts of Guangdong province in China, preserved in 2.5% potassium dichromate, and stored at −20°C for use.

### 2.2. Genomic DNA Extraction

The adult hookworms preserved were repeatedly washed with double-distilled water (ddH_2_O), and total genomic DNA was extracted using the Wizard® SV Genomic DNA Purification System (Promega, Guangzhou, China) according to the manufacturer's protocols. All clinical fecal samples were dissolved in ddH_2_O and then pretreated with 5 cycles of heating at 100°C for 5 min, followed by immediate freezing at −80°C for 5 min. Genomic DNA of clinical fecal samples was extracted using Stool DNA extraction kit (Omega, Guangzhou, China) according to the manufacturer's protocols. Extracted DNAs were then stored at −20°C for use.

### 2.3. PCR Amplification of ITS1 Sequence

The ITS1 sequences of* A. ceylanicum* and* A. caninum* were amplified using a forward primer AF (5′-CTTTGTCGGGAAGGTTGG-3′) and a reverse primer AR (5′-TTCACCACTCTAAGCGTCT-3′) designed by Liu et al. [15]. The predictive amplification fragment was 404 bp. Polymerase chain reactions were performed in 25 *μ*L, including 12.5 *μ*L of Premix Ex-Taq polymerase (TaKaRa, Dalian, China), 9.5 *μ*L of ddH_2_O, 0.5 *μ*L of each primer AF/AR (50 *μ*mol/L), and 2 *μ*L of DNA sample. PCR cycling parameters were as follows: 1 cycle at 94°C for 5 min and then 35 cycles at 94°C for 30 sec, at 61.5°C for 30 sec, and at 72°C for 45 sec, followed by 1 cycle at 72°C for 7 min. The PCR products were analysed by gel electrophoresis in 1.5% agarose gels, stained with 0.2 mg/ml ethidium bromide, and visualized on a UV transilluminator.

### 2.4. Preparation of Standard Plasmids

The purified PCR products were cloned in* Escherichia coli* and connected with pMD18-T (TakaRa, Dalian, China) and then transferred into DH5*α* Competent Cells (TaKaRa, Dalian, China). Positive clones were screened by bacterial PCR and sent to Shenggong Corporation (Shanghai, China) for sequencing. The plasmid DNAs were extracted using the Plasmid Kit (Omega, Guangzhou, China). These plasmids containing ITS1 sequence of* A. ceylanicum* and* A. caninum* were named AceP and AcaP, respectively. The A260/A280 value and concentration of plasmids (1 *μ*L) were measured using ultra-micro-spectrophotometer, where A260/A280 value and optimal concentration should be 1.8~2.0 and 50 ng/*μ*L. These plasmids were stored at −20°C for use.

### 2.5. Establishment of *T*_m_-Shift Method Based on SNP

Three sets of *T*_m_-shift primers (including two forward specific primers and one common reverse primer) based on three SNPs (ITS71, ITS197, and ITS296) were designed by software Primer Premier 5.0, referencing to two sequences (KM066110.1, LC177192.1) downloaded from GenBank. Their sequence composition and predictive amplification fragment are shown in Table 1. The above-mentioned primers were synthesized by Shenggong Corporation (Shanghai, China), diluted with sterile double-distilled water at a final concentration of 10 pmol/*μ*L, and stored at −20°C for use. PCR amplification and *T*_m_-shift reaction were performed once in Rotor-Gene Q. The reaction system was performed in 20 *μ*L, including 10 *μ*L of SYBR®Premix Ex-Taq™ II (2x), 0.4 *μ*L of long-tail primer, 0.4 *μ*L of short-tail primer, 0.8 *μ*L of common reverse primer, 7.4 *μ*L of ddH_2_O, and 1.0 *μ*L of plasmid. PCR cycling parameters were as follows: initial denaturation at 95°C for 5 min and then 40 cycles at 95°C for 10 sec and 63°C for 30 sec. Melting process was from 70 to 95°C at the rate of 0.5°C/sec.

### 2.6. Stability, Sensitivity, and Accuracy Test

The detection effect of the *T*_m_-shift was assessed through the stability, sensitivity, and accuracy test. We tested the reproducibility of two known standard plasmids (AceP and AcaP). Intra-assays were tested seven times, and interassays were tested three times for each plasmid. For detection of the sensitivity of the *T*_m_-shift method, two standard plasmids (AceP and AcaP) were diluted ten times and detected according to the concentration of 1 : 10~1 : 10^8^. To evaluate the *T*_m_-shift method's accuracy, we detected ten samples with known hookworm species. All reaction system and cycling parameters were the same as mentioned above.

### 2.7. Clinical Detection

Fifty fecal samples from the stray dogs were examined by the flotation technique with saturated zinc sulfate and the *T*_m_-shift method. *T*_m_-shift classification results were further confirmed by DNA sequencing.

## 3. Results

### 3.1. Amplification of ITS1 Fragment and Preparation of Plasmids

**Figure 1:**
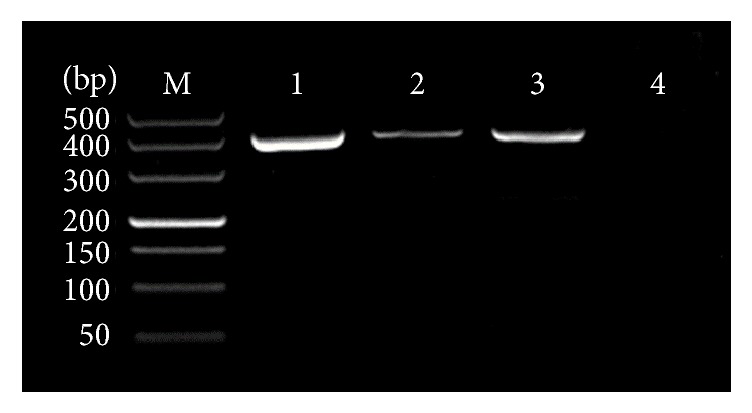
PCR amplification of the ITS1 sequence of two hookworms. MDL-500 DNA marker; 1: positive control; 2:* A. ceylanicum*; 3:* A. caninum*; 4: negative control.

The amplified ITS1 fragment from two genomic DNAs of dog-derived* A. ceylanicum* and* A. caninum* was 404 bp long (Figure 1) and the generated sequence data were submitted to GenBank (accession numbers: MG733994, MG733993). BLAST analysis indicated the highest similarity (100%) with* A. ceylanicum* from Japan (LC036567) and the highest similarity (99%) with* A. caninum *from Australia (KP844730). Thus, two hookworms were identified as* A. ceylanicum *and* A. caninum*. The A260/A280 values of positive plasmids AceP and AcaP were between 1.8 and 2.0, respectively. Their concentrations were 53.3 ng/*μ*L and 50.3 ng/*μ*L, respectively.

### 3.2. Detection of qPCR-*T*_m_-Shift

**Figure 2:**
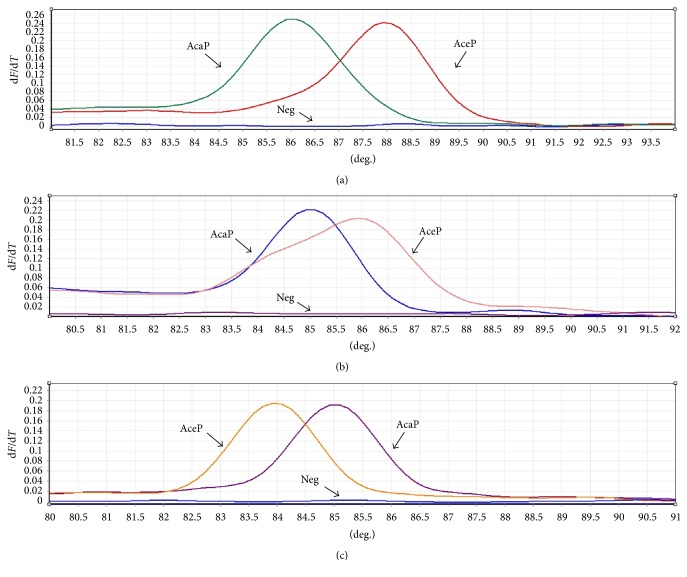
Standard curves of *T*_m_-shift for two hookworm standard plasmids based on ITS71 (a), ITS197 (b), and ITS296 (c). AceP:* A. ceylanicum* standard plasmid; AcaP:* A. caninum* standard plasmid; Neg: negative control.

The standard curves of *T*_m_-shift based on ITS71, ITS197, and ITS296 for standard plasmid AceP and AcaP are shown in Figure 2. The result showed that *T*_m_-shift method based on three SNPs could distinguish between* A. ceylanicum* and* A. caninum*. Software analysis showed that the *T*_m_ values of AceP (*A. ceylanicum*) and AcaP (*A. caninum*) in three sets of primers were 88.0°C and 86.0°C (ITS71), 86.0°C and 85.0°C (ITS197), and 84.0°C and 85.0°C (ITS296), respectively.

### 3.3. Stability, Sensitivity, and Accuracy

The stability test results are shown in Table 2. In primer ITS71, the coefficient of variation (CV) of AceP and AcaP melting temperature (*T*_m_) was 0.15 and 0.09%. In primer ITS197, the CV of AceP and AcaP melting temperature was 0.14 and 0.18%. And in primer ITS296, the CV of AceP and AcaP melting temperature was 0.07 and 0.13%. When AceP and AcaP samples were diluted to 1 : 10^7^ (5.33 × 10^−6^ ng/*μ*L and 5.03 × 10^−6^ ng/*μ*L), the *T*_m_-shift method was still able to identify both of them. However, when the samples were diluted to 1 : 10^8^, all of them could not be determined (Table 3). A total of ten known hookworm samples from dogs were randomly selected and detected. The *T*_m_-shift detection results based on ITS71, ITS197, and ITS296 were identical to their known hookworm species (Table 4).

### 3.4. Clinical Detection

**Figure 3:**
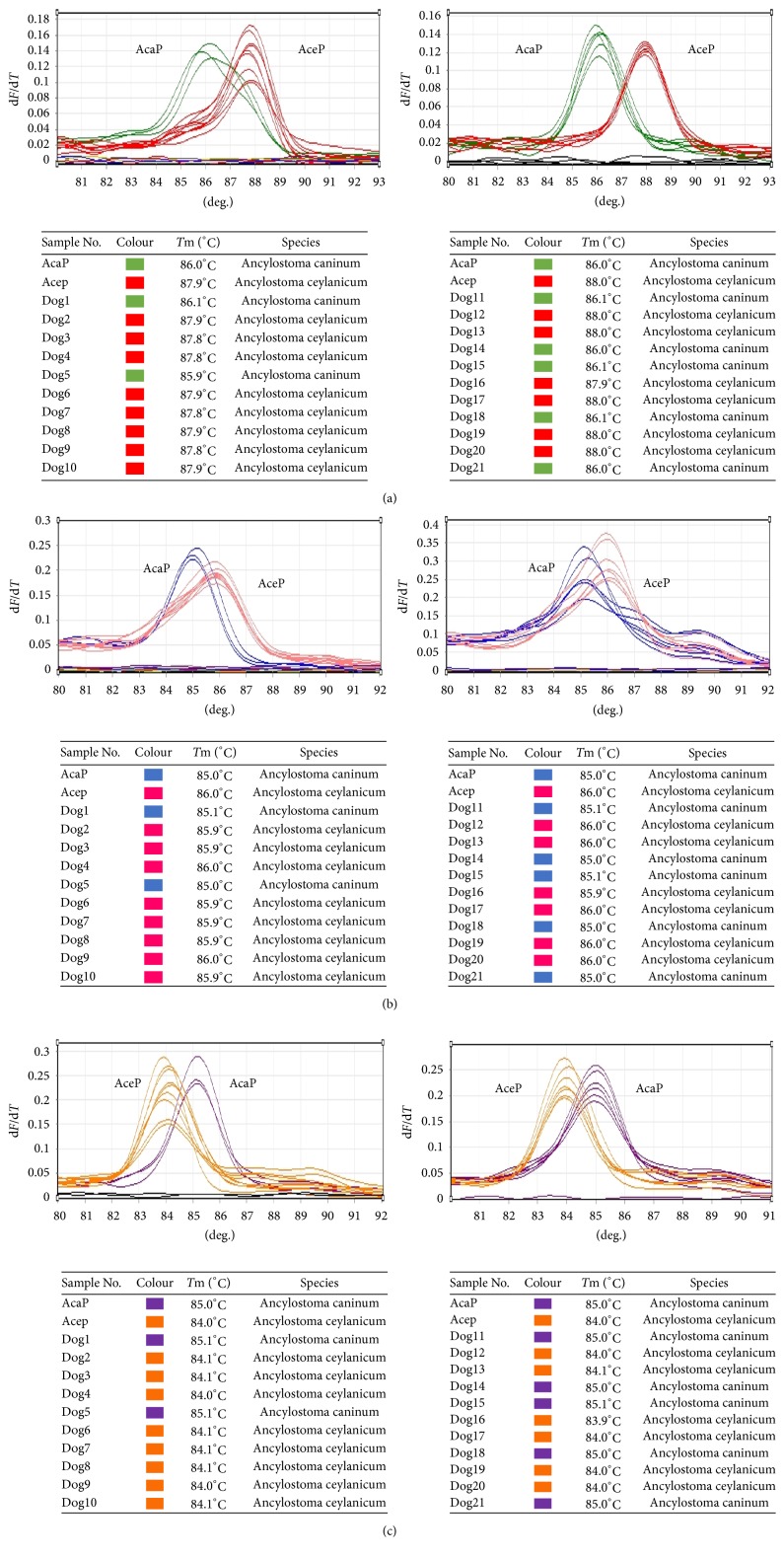
Melting curves of *T*_m_-shift based on ITS71 (a), ITS197 (b), and ITS296 (c) and detection results for 21 hookworm-positive samples.

Seventeen out of fifty fecal samples from dogs were microscopically positive for hookworm but twenty-one of them were positive for hookworm using *T*_m_-shift method, where fourteen were identified as* A. ceylanicum* and seven as* A. caninum*. The melting curves and detection results are shown in Figure 3. All detection results were identical to their sequencing results.

## 4. Discussion

Dogs are important reservoir host to zoonotic hookworms* A. ceylanicum* and* A. caninum. *Genetic evolution analysis indicated that dog-derived* A. ceylanicum* and human-derived* A. ceylanicum *from China [25], Malaysia [26], Kampuchea [27], and India [13] have the closest genetic relationship, and the infection rate of* A. ceylanicum *between humans and dogs had a strong correlation in the same region, as well as confirming the possibility of transmission of* A. ceylanicum* between humans and animals [28].* A. caninum* mainly infects dogs and cats and sometimes infects people. Dogs are both companion animals for humans and susceptible animals to zoonotic hookworms. With the development of economy and improvement of life quality, the number of people raising dogs has been increasing. Therefore, the establishment of *T*_m_-shift method for detection of* A. ceylanicum* and* A. caninum* has important significance in the prevention and control of zoonotic ancylostomiasis.

Currently, the species identification of hookworm is mainly dependent on molecular means such as specific PCR, multi-PCR, RFLP, and HRM [14–16]. However, there are still some limitations. The conventional PCR can only detect a single pathogen. Although multi-PCR can simultaneously detect multiple pathogens, it is necessary to design more primers and the PCR conditions are demanding. The RFLP technology can only select specific endonuclease, and the operation is tedious and time-consuming, with low sensitivity for false negatives. HRM is similar to the *T*_m_-shift method through test of the melting curve of PCR products. But it is based on the different GC content and base complementarity in the same DNA sequence to detect samples. When difference bases are counteracted in the sequence, two peaks of melting curve are difficult to distinguish and easily cause detection failure [29]. Compared with specific PCR, multi-PCR, and RFLP, the *T*_m_-shift method can detect 36 or 72 samples simultaneously. Its closed tube operation can greatly reduce the risk of contaminated samples. Also, there is no need to use the poisonous reagent ethidium bromide (EB), which thus can reduce pollution to the environment and ensure safety of the researchers. The *T*_m_-shift method has clear price advantage compared with HRM, because of the use of instruments for ordinary fluorescence PCR and fluorescent dyes for ordinary SYBR Green I rather than saturation dyes Eva Green and LC Green. In addition, the detection efficiency is improved through amplification of the target fragment by two-step method.

Although the *T*_m_-shift detection method has its unique advantages, it also has some limitations. It has higher demand of two specific primers, whose *T*_m_ value should be 59~62°C, and their length should be controlled in 15~22 bp. In order to give better amplification efficiency of Taq polymerase, the gap of the *T*_m_ values between the reverse primer and the specific primers (before joining at the 5′-end sequence) must be maintained between 4 and 5°C. It is due to the high screening conditions of specific primers that some SNP loci failed to design suitable primers. Among five SNP loci (Table 1) originally designed in this experiment, two loci (ITS26 and ITS48) failed to distinguish between* A. ceylanicum* and* A. caninum*, thus leading to the failure of detection. Another three SNP loci (ITS71, ITS197, and ITS296) could distinguish between two species of hookworms. The *T*_m_ value of the melting curve between* A. ceylanicum* and* A. caninum* differed at 2°C on ITS71 but 1°C on ITS197 and ITS296. The ITS71 locus identification had the best effect. Among 50 dog-derived fecal samples detected, 21 samples were positive for hookworms by *T*_m_-shift method (14* A. ceylanicum *and 7* A. caninum*), which had 4 more positive samples than microscopic examination (17 positive samples), and can accurately identify the species of hookworms from stray dogs.

In conclusion, *T*_m_-shift method based on three SNP loci of ITS1 sequences of* A. ceylanicum *and* A. caninum* was used for the first time for their classification. The successful identification of two hookworms indicates that the method is specific, sensitive, economical, practical, and safe, which can provide a new technical means for clinical detection of hookworm and zoonotic risk assessment.

## Figures and Tables

**Table 1:** Primers for *T*_m_-shift method based on five SNPs.

Primer	Nucleotide sequence (5′–3′)	Product length (bp)
ITS71TF(Aca)	**GCGGCG**CTATGTGCAGCAAGAGT	
ITS71CF(Ace)	**GCGGGCAGGGCGGG**CTATGTGCAGCAAGAGC	113
ITS71R	ACAAGCAGTAAGGCGGCATTCA	
ITS197GF(Aca)	**GCGGCG**TGAGCATTAGGCTAACGCCTG	
ITS197AF(Ace)	**GCGGGCAGGGCGGG**TGAGCATTAGGCTAACGCCTA	114
ITS197R	ACGATTCTGCAAACATTAAACGTAAAAAGT	
ITS296TF(Ace)	**GCGGCG**TTTGCAGAATCGTGACTTT	
ITS296GF(Aca)	**GCGGGCAGGGCGGG**TTTGCAGAATCGTGACTTG	127
ITS296R	TTCACCACTCTAAGCGTCT	
ITS26AF(Aca)	**GCGGCG**GAAGGTTGGGAGTATCA	
ITS26GF(Ace)	**GCGGGCAGGGCGGG**GAAGGTTGGGAGTATCG	121
ITS26R	GTCTAAAGCTCAGCGAAAC	
ITS48AF(Aca)	**GCGGCG**CCGTTACAGCCCTACGTA	
ITS48GF(Ace)	**GCGGGCAGGGCGGG**CCGTTACAGCCCTACGTG	116
ITS48R	ATGCAATGCTCATCAAGTC	

The GC tails are bold and underlined. Primers at ITS26 and ITS48 failed to distinguish between *A. ceylanicum* and *A. caninum*.

**Table 2:** The stability of *T*_m_-shift method.

Repeat	ITS71 (*T*_m_)		ITS197 (*T*_m_)		ITS296 (*T*_m_)	
	AcaP	AceP	AcaP	AceP	AcaP	AceP
First (*n* = 7)	86.1 ± 0.1°C	88.0 ± 0.1°C	85.1 ± 0.1°C	85.9 ± 0.1°C	85.0 ± 0.1°C	84.0 ± 0.1°C
Second (*n* = 7)	86.1 ± 0.2°C	88.1 ± 0.1°C	85.1 ± 0.1°C	85.9 ± 0.1°C	85.0 ± 0.1°C	84.0 ± 0.2°C
Third (*n* = 7)	86.1 ± 0.1°C	87.9 ± 0.1°C	85.1 ± 0.2°C	85.9 ± 0.1°C	85.1 ± 0.1°C	84.1 ± 0.1°C
Average (*n* = 21)	86.10°C	88.00°C	85.10°C	85.90°C	85.03°C	84.03°C
CV	0.15%	0.09%	0.14%	0.18%	0.07%	0.13%

**Table 3:** The sensitivity of *T*m-shift method.

Dilution	ITS71 (*T*_m_)		ITS197 (*T*_m_)		ITS296 (*T*_m_)	
	AcaP	AceP	AcaP	AceP	AcaP	AceP
1 : 10^1^	86.1°C	88.0°C	85.0°C	86.0°C	85.0°C	83.9°C
1 : 10^2^	85.9°C	88.1°C	85.0°C	86.0°C	85.0°C	84.0°C
1 : 10^3^	86.0°C	88.1°C	85.1°C	86.0°C	85.0°C	84.0°C
1 : 10^4^	86.0°C	88.0°C	85.0°C	86.0°C	85.0°C	84.0°C
1 : 10^5^	86.1°C	88.1°C	84.9°C	86.1°C	85.0°C	83.9°C
1 : 10^6^	86.1°C	88.0°C	85.1°C	86.1°C	84.9°C	83.9°C
1 : 10^7^	86.0°C	88.0°C	85.1°C	86.1°C	85.0°C	83.9°C
1 : 10^8^	—	—	—	—	—	—

—: not detected.

**Table 4:** The accuracy of *T*_m_-shift method.

Sample	Species	ITS71 (*T*_m_)	ITS197 (*T*_m_)	ITS296 (*T*_m_)	GenBank
D22	*A. caninum*	86.0°C	85.0°C	85.0°C	KC755016
D55	*A. ceylanicum*	88.0°C	86.0°C	84.0°C	KC755015
D60	*A. ceylanicum*	88.0°C	85.9°C	83.9°C	KC755020
D74	*A. ceylanicum*	88.0°C	86.0°C	84.0°C	KC755021
D23	*A. caninum*	86.1°C	85.0°C	85.1°C	KC755017
D28	*A. caninum*	86.0°C	85.1°C	85.0°C	KC755018
D34	*A. caninum*	86.1°C	85.1°C	85.0°C	KC755019
D79	*A. ceylanicum*	88.0°C	86.0°C	84.0°C	KC755022
D23	*A. ceylanicum*	87.9°C	86.0°C	84.0°C	KF279132
D32	*A. ceylanicum*	88.0°C	86.0°C	84.0°C	KF279133

Samples D22, D23, D28, D34, D55, D60, D74, and D79 were isolated and identified by Liu et al. [2]. Samples G23 and G32 were isolated and identified by Liu et al. [6].
